# Supplementary material for: Identification of Two Subsets of Subcompartment A1 Associated with High Transcriptional Activity and Frequent Loop Extrusion
Source: Biology (Basel). 2023 Jul 27;12(8):1058. doi: 10.3390/biology12081058 (PMC10451812; doi:10.3390/biology12081058)
Supplement: Supplementary file 1 [file biology-12-01058-s001.zip › Supplementary Figures.pdf]

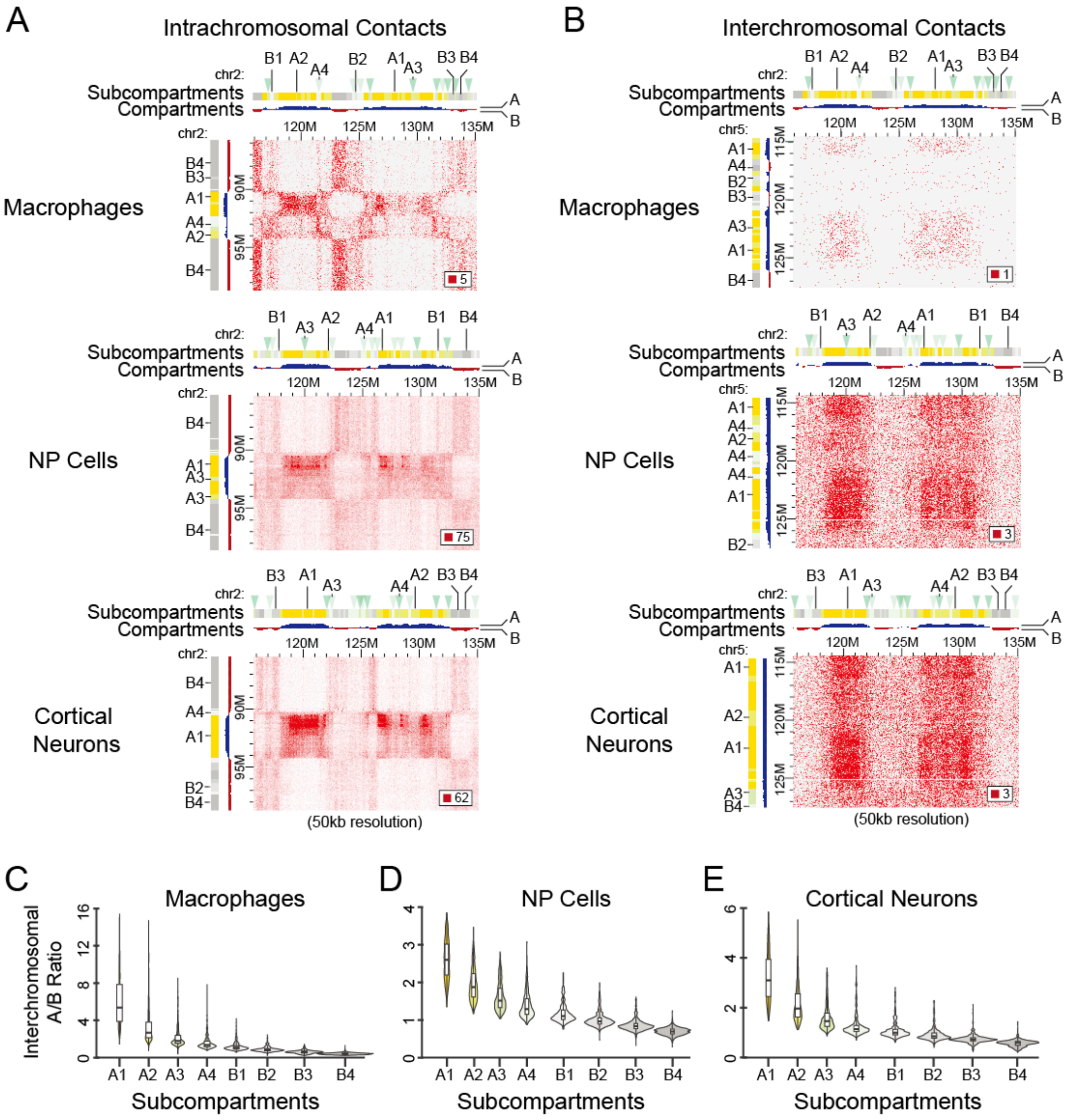

Figure S1. The relationship between Hi-C contacts and subcompartments. A) Heatmaps showing intrachromosomal (left panel) and interchromosomal (right panel) chromatin contacts in macrophages (upper panel), neural progenitor cells (middle panel) and thymocytes (lower panel) respectively. B) Violin plots showing the interchromosomal A/B ratio of eight sub-compartments respectively in macrophages (left panel), neural progenitor cells (middle panel) or thymocytes (right panel).

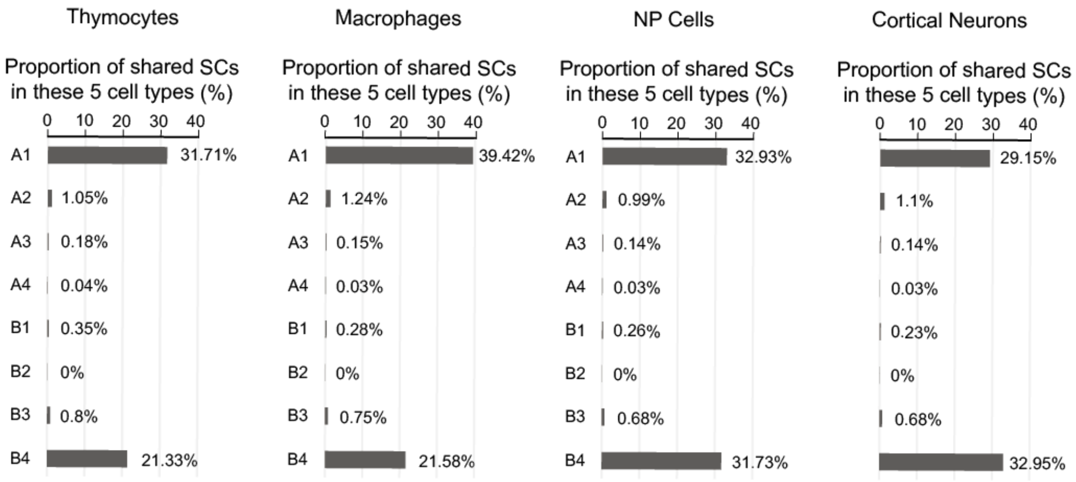

Figure S2. Identification of the same subcompartment annotation in different cell types, including thymocytes, macrophages, neural progenitor cells and cortical neurons separately.

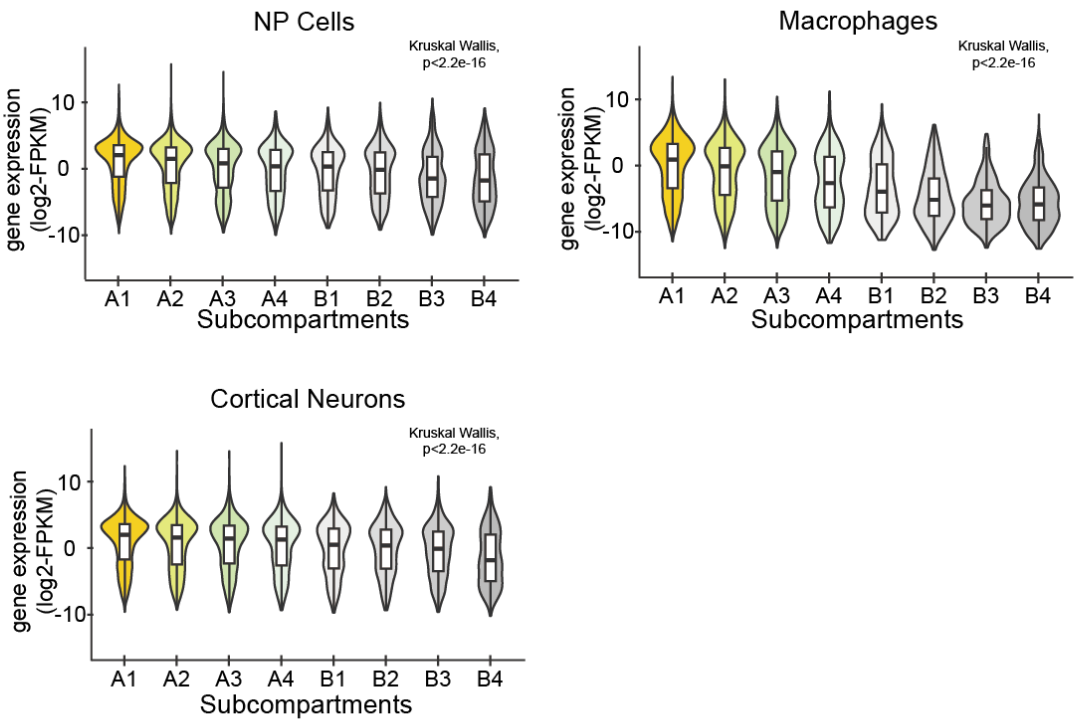

Figure S3. The correlation between gene expression levels and subcompartment positioning. Violin plots showing the gene expression levels of total protein-coding genes in eight different subcompartment types in neural progenitor cells, macrophages and cortical neurons separately. FPKM, fragment per kilo-base exon per million mapped reads.

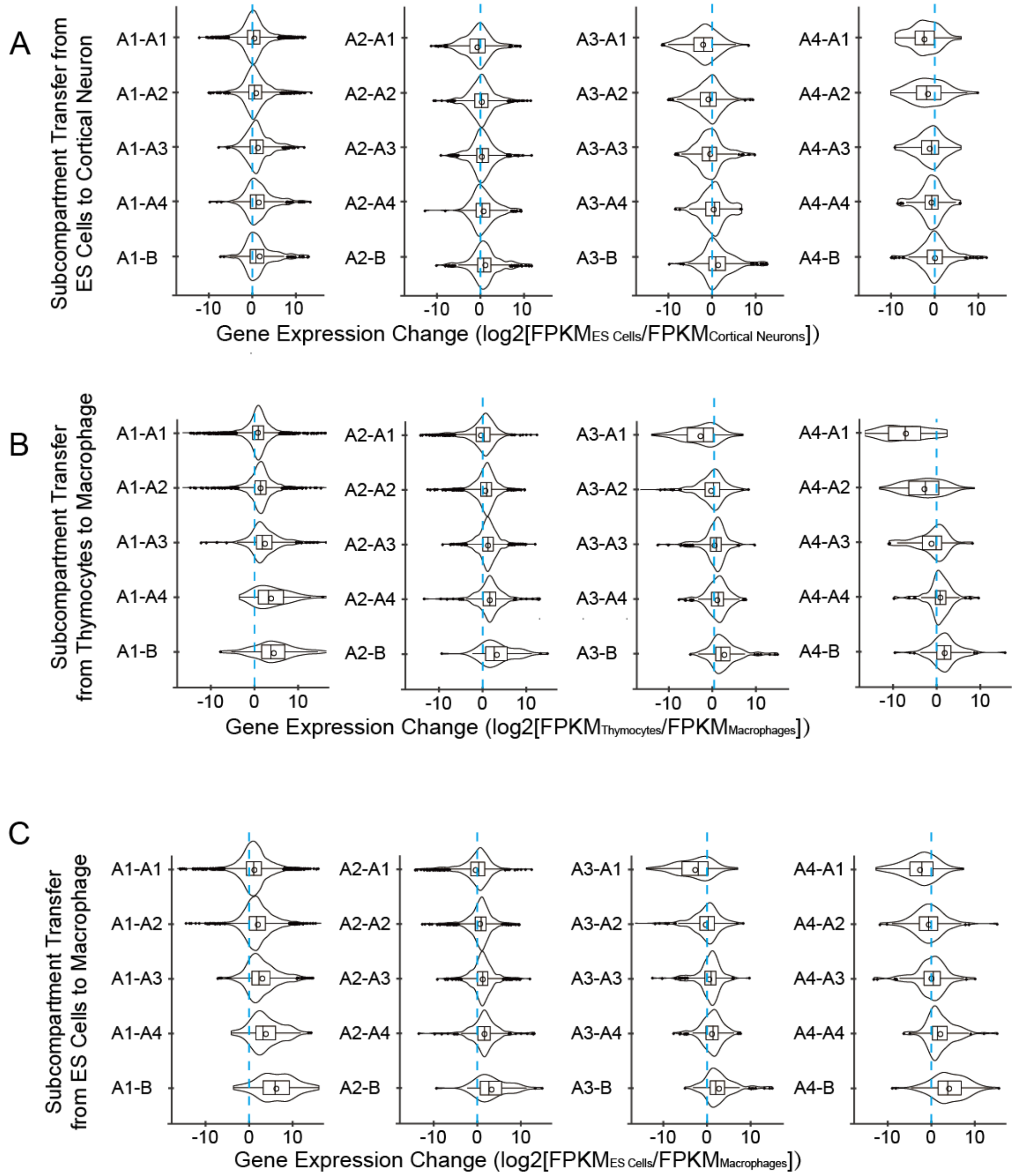

Figure S4. Relation between subcompartment repositioning and changes of gene expression from embryonic stem cells to cortical neuron (A) or from thymocytes to macrophages (B) or from embryonic stem cells to macrophages (C) for total genes.

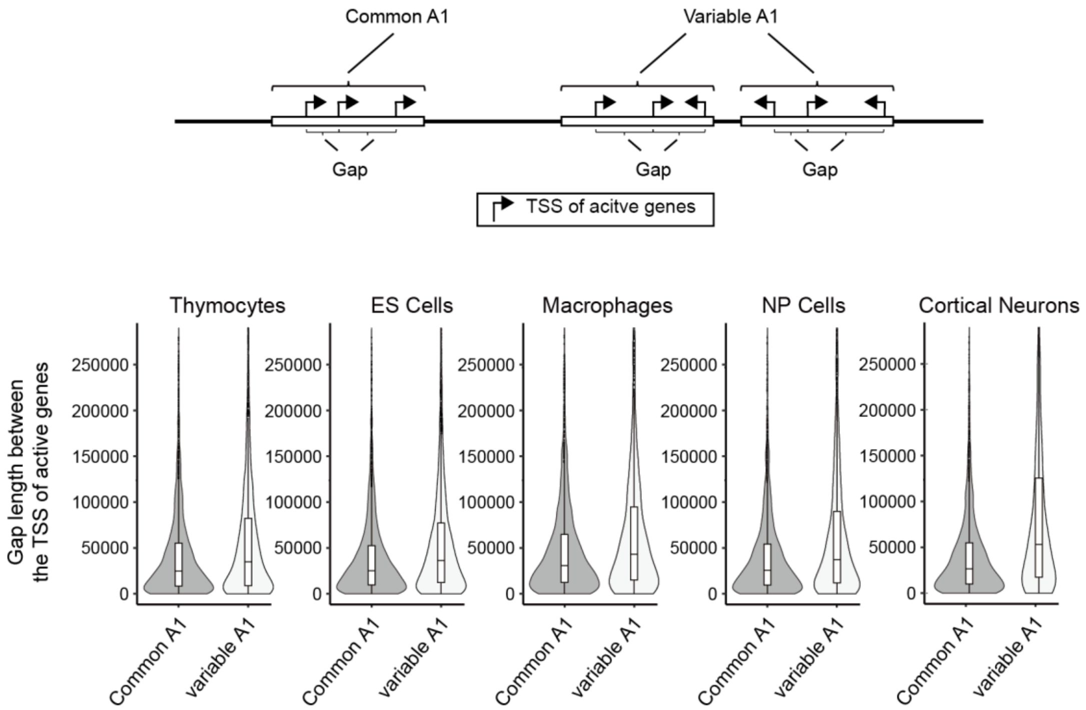

Figure S5. Gene density in common A1 and variable A1. Diagram showing the length of gaps between transcription start sites (TSS) of active genes. The violin plots display all gap lengths in common A1 (dark grey) or variable A1 (light grey).

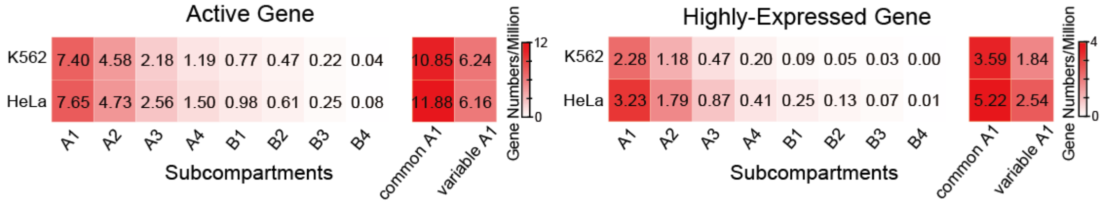

Figure S6. Gene density of different subcompartments in Hela and K562 cells. A) Gene numbers per million of active genes (upper) or highly expressed genes (lower) in each subcompartment type. B) Size of common A1 subcompartments, total A1 sub-compartments and total A compartments in five different cell types.

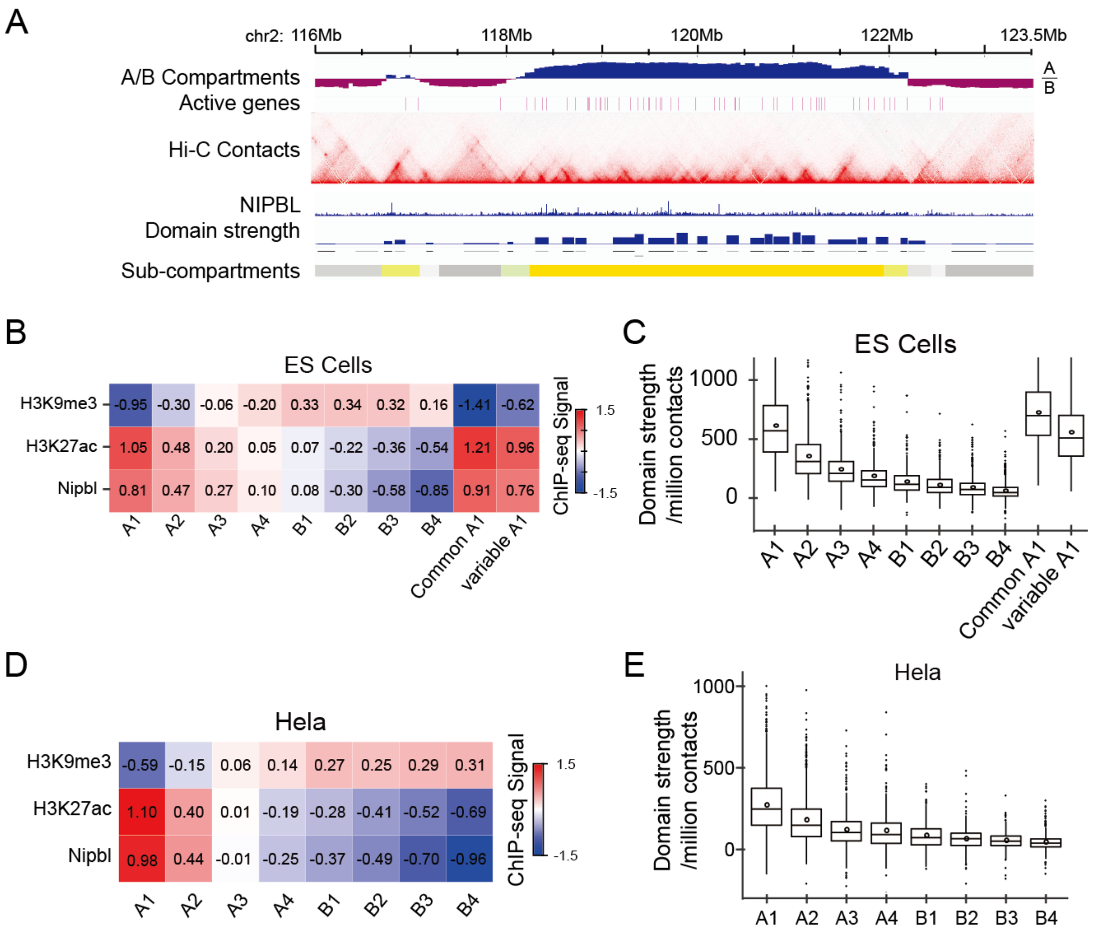

Figure S7. Contact domain strength in in both mouse ES cells and human HeLa cells. A) Hi-C contact maps in a region on chromosome 2 (chr2: 116,000,000-123,500,000) showing contact domains in ES cells at 5-kb resolution aligned to the annotated tracks of A/B compartments, NIPBL occupancy, domain strength, and subcompartments. Transcription start sites of active genes are indicated in purple lines. B) Enrichment analysis of histone modification H3K9me3 and H3K27ac, as well as NIPBL signals in each subcompartment type or each subtype of subcompartment A1 across genome in ES cells. C) Box plot showing contact domain strength in eight subcompartments and two A1 subsets. D) Enrichment analysis of histone modification H3K9me3 and H3K27ac, as well as NIPBL signals in each subcompartment type in the human Hela cells. E) Box plot showing contact domain strength in eight subcompartments.
