# Supplementary material for: Identification of Two Subsets of Subcompartment A1 Associated with High Transcriptional Activity and Frequent Loop Extrusion
Source: Biology (Basel). 2023 Jul 27;12(8):1058. doi: 10.3390/biology12081058 (PMC10451812; doi:10.3390/biology12081058)
Supplement: Supplementary file 1 [file biology-12-01058-s001.zip › Table S1. High throughput Sequencing Datasets Used in The Study.pdf]

Table S1. High throughput Sequencing Datasets Used in The Study

Data types	Source from	Access number
Hi-C, RNA-seq data (ES Cells, Primary Neocortex Cortical Neurons, Primary Neocortex NP Cells)	Bonev et al., 2017	GEO：GSE96107
RNA-seq data (Primary Thymocytes, $CD69^{-}CD4^{+}CD8^{+}$)	Karimi et al., 2021	GEO：GSE154670
RNA-seq data (Macrophages)	Cuartero et al.2018	GEO：GSE108599
RNA-seq data (K562, HeLa)	She et al., 2023	GEO：GSE207330
Hi-C, H3K27ac ChIP-seq data (Primary Thymocytes, $CD69^{-}CD4^{+}CD8^{+}$)	Guo et al., 2022	GEO：GSE199059
Hi-C data (Macrophages)	Mumbach et al., 2019	GEO：GSE115524
Hi-C data (K562)	https://data.4dnucleome.org/	4DN：4DNFICEGAHRC
Hi-C data (HeLa)	https://data.4dnucleome.org/	4DN：4DNFITUOMFUQ
CUT&Tag, ChIP-seq data (Nipbl for K562 and HeLa, H3K27ac for K562)	This Study	GEO: GSE227868
ChIP-seq data (H3K9me3 for ES Cells)	Navarro et al., 2020	GEO: GSE149080
ChIP-seq data (H3K27ac for ES Cells)	Kubo et al., 2021	GEO: GSE160890
ChIP-seq data (Nipbl for ES Cells)	Kagey et al., 2010	GEO: GSE22557
ChIP-seq data (Nipbl for thymocyte)	Seitan et al., 2013	GEO: GSE48763
ChIP-seq data (H3K9me3 for HeLa)	Timms et al., 2016	GEO: GSE86814
ChIP-seq data (H3K27ac for HeLa)	ENCODE Project Consortium (2012).	GEO: GSE29611
ChIP-seq data (H3K9me3 for K562)	Siegenfeld et al., 2022	GEO: GSE198613
